# Prevalence of intestinal parasites and the absence of soil-transmitted helminths in Añatuya, Santiago del Estero, Argentina

**DOI:** 10.1186/s13071-018-3232-7

**Published:** 2018-12-14

**Authors:** Maria Victoria Periago, Rocío García, Osvaldo Germán Astudillo, Marta Cabrera, Marcelo Claudio Abril

**Affiliations:** 1 Consejo Nacional de Investigaciones Científica y Técnicas (CONICET), Fundación Mundo Sano, Paraguay, 1535 Buenos Aires, Argentina; 2 Fundación Mundo Sano, Paraguay, 1535 Buenos Aires, Argentina; 3 Instituto Nacional de Enfermedades Infecciosas, Administración Nacional de Laboratorios e Institutos de Salud “Dr. Carlos G. Malbrán”, Av. Vélez Sarsfield, 563 Buenos Aires, Argentina

**Keywords:** Soil-transmitted helminths, Intestinal parasites, Añatuya, Santiago del Estero, Argentina

## Abstract

**Background:**

Intestinal parasites (IP) have been reported in point studies from different provinces of Argentina. The presence of soil-transmitted helminths (STH) was detected in many of these studies, including varied prevalences of all five species of STH in the north were the climate is more appropriate for transmission. Nonetheless, Argentina lacks a comprehensive prevalence map of STH. Therefore, the objective of this study was to determine the prevalence of intestinal parasites, focusing on STH, in rural and peri-urban areas of Añatuya, Santiago del Estero Province and identifying risk factors for their transmission.

**Methods:**

We conducted a cross-sectional survey in the entire population of three rural lots located on the outskirts of the city between March and June of 2015 and among children in a peri-urban neighbourhood of Añatuya city in July 2016. Socio-economic variables, characteristics of the house and stool samples were collected from each household.

**Results:**

A total of 470 stool samples were analysed. The prevalence of STH was extremely low, with only 2 people being positive for hookworm and no detection of other STH. The prevalence of IP was 11.7% for protozoans and 11.1% for helminths. IP were significantly more prevalent in pre-school and school-aged children than in adults (*P* < 0.05). The level of education of adults was also significantly associated with infection (*P* = 0.001), as well as the practice of informal slaughter of animals (*P* = 0.002) and the presence of unimproved walls (*P* = 0.046) and unimproved floors (*P* = 0.021) in the household. Nonetheless, the only significant predictors of IP in the logistic regression analysis were age (*P* < 0.001) and main source of electricity (*P* = 0.026).

**Conclusions:**

The total prevalence of intestinal parasites in the study population was 22.6%. The intestinal parasites detected are transmitted mostly through water (*Giardia lamblia*) and close contact (*Hymenolepis nana*), evidencing the need to improve quality control in water facilities and access to improved sanitation to avoid contamination of stored water. The presence of IP was significantly associated with age (with children being more susceptible), households containing unimproved walls and those that did not have access to an electricity network.

## Background

Intestinal parasites (IP) are a group of cosmopolitan parasites that include protozoan and helminth species. These IP, especially some protozoans, affect both rural and urban human populations. Moreover, some species are more prevalent in populations with poor access to water, sanitation and hygiene (WASH), including soil-transmitted helminths (STH) [1]. The different species of STH, *Ascaris lumbricoides*, *Trichuris trichiura* and hookworms (*Ancylostoma duodenale* and *Necator americanus*) are endemic in tropical and subtropical areas of the world and are included in the list of the 20 neglected tropical diseases (NTDs) recently updated by the World Health Organization (WHO) [2]. Although *Strongyloides stercoralis* is a STH, it is not included in this group because of its specific characteristics with respect to diagnosis, quantification and treatment [3]. These diseases have a high burden that affect millions of people worldwide. Disability-adjusted life years (DALYs) in Latin America and the Caribbean (LAC) are 60,000 for *Ascaris*, 73,000 for *Trichuris* and 20,000 for hookworms [4]. An estimation for *S. stercoralis* is not available due to the difficulty in its diagnosis, with the need of specific techniques [3].

The prevalence of IP in the population is tied to various factors. Studies conducted in urban, peri-urban and rural population of northern Argentina and other parts of the world, observed different prevalences of IP depending on socioeconomic status, sanitary and environmental conditions and access to water [5–9]. This is mainly due to the transmission route of several IP, through soil and food contamination as well as faecal contamination of water used for drinking and cooking. Moreover, other conditions like overcrowding, malnutrition, lack of hygiene habits and waste management and treatment also influence prevalence [10–16]. Most of the studies conducted in Argentina have focused on school-aged populations; individuals infected with protozoans (*Giardia intestinalis*, *Blastocystis hominis* and amoebas), helminths (*Enterobius vermicularis* and *Hymenolepis nana*) and STH were detected in both rural and peri-urban areas. The most prevalent parasites detected were *G. intestinalis* [10, 17, 18], *B. hominis* [5, 6, 8, 11, 18–21], *E. vermicularis* [5, 11, 22–24], *A. lumbricoides* [10, 18, 23] and *T. trichiura* [23] all of which are transmitted through the faecal-oral route. High prevalences of STH species transmitted through skin penetration have also been detected [21, 25, 26].

The current treatment and control of STH in endemic areas provide a good strategy for the control of morbidity, but are not enough to interrupt transmission, which would require improvements in water, sanitation and hygiene (WASH). Even though resolution WHA54.19 from the World Health Assembly (WHA) of the WHO specific for STH, urges member states to “promote access to safe water, sanitation and health education through intersectoral collaboration” [27], little has been done to date. In most endemic countries, control measures are based on mass drug administration (MDA) of a single dose of either albendazole (400 mg) or mebendazole (500 mg) in school aged children (SAC).

STH are endemic in the northeast and northwest regions of Argentina and estimates suggest there are around 6.5 million infected [23]. It is important to note that within those infected by STH, *Strongyloides stercolaris* is included, with important prevalences in some areas of Argentina [28] as well as in other parts of the world [29]. The presence of all five species of STH has been detected in different provinces located in the north of the country including Corrientes [11, 20], Misiones [24, 26], Salta [18, 25, 30, 31] and Tucumán [6, 18].

The National Ministry of Health partially implemented a control programme for STH in the past which ran for only two years (2005–2007) and was based on MDA with mebendazole. Due to its short duration and a lack of baseline prevalence of STH in the country, the impact of the programme could not be assessed. A recent systematic review of the literature [28] has shed some light on the prevalence of these parasites in Argentina, but a complete map of the situation is far from being achieved.

In this context, the objective of this study was to determine the prevalence of intestinal parasites, with a focus on STH, in rural and peri-urban areas of Añatuya, Santiago del Estero, Argentina, and to identify risk factors for their transmission.

## Methods

### Study area and study population

**Fig. 1:**
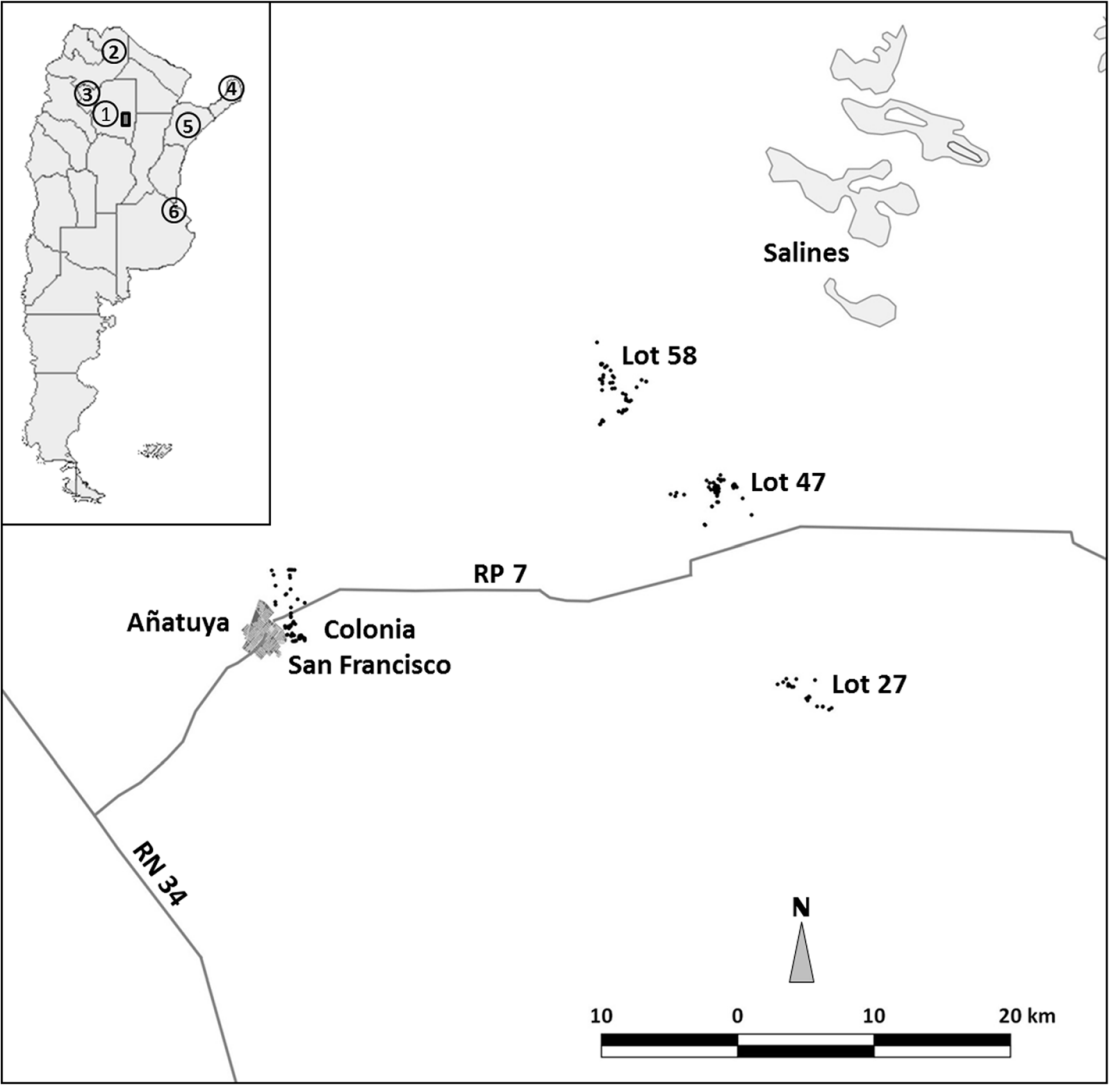
Map of the study area, Añatuya, Santiago del Estero, Argentina. Each point represents the individual georeferenced houses that were included in the study. *Key*: 1, Province of Santiago del Estero; 2, Province of Salta; 3, Province of Tucumán; 4, Province of Misiones; 5, Province of Corrientes; 6, City of Buenos Aires. The map was created using GvSig 1.11 software (Asociación gvSIG, 2017)

The cross-sectional study was conducted in two stages, the first in three rural settlements in the Department of General Taboada between March and June of 2015, and the second in a peri-urban neighbourhood of the City of Añatuya (Fig. 1) during the month of July (2016). This study was originally designed as a community-wide intervention in rural settlements with the aim of identifying the presence of STH and the settlements were selected due to the presence of living conditions appropriate for the transmission of such parasites. Given the very low prevalence of STH found in these rural communities, the study was extended to a semi-urban neighbourhood of Añatuya to confirm the results obtained. In order to maximize the chances of finding STH and corroborating the results from the rural settlements, only children from 1 to 15 years-old were selected.

Añatuya is located within the Department of General Taboada in the Province of Santiago del Estero in the north of Argentina. It is part of the Great Chaco ecoregion. More specifically, Añatuya is located in the semiarid sub-region of the Great Chaco [32] approximately 7 km from the Salado River (“salty river”). The sediments around this area are thin and not very permeable, with subterranean water located at less than 3 m deep. Therefore, direct evaporation and evapotranspiration of the vegetation cover leads to salt accumulation, making it hard to obtain potable water [32]. Moreover, the presence of arsenic is common and the lack of rain in the area does not favour the regeneration of potable water aquifers. Santiago del Estero has two main seasons, hot and rainy from October to March, and dry and moderate from April to September. The dry season is critical due to the lack of humidity of the soil (with 55 to 120 mm of rain, an average of 78 mm and only 8.5 days of rain) during the entire period. This creates a deficit between the amount of water that evaporates and rainfall [32]. The median annual temperature for the Province of Santiago del Estero is 21.5 °C, although there is a wide range throughout the year (10–47 °C). The average temperature in the winter is between 15–20 °C and between 26–28 °C in the summer. The population density in this region is low, with 874,006 inhabitants in the entire province, 38,000 in the Department of General Taboada and 23,286 inhabitants in the City of Añatuya [30].

**Fig. 2:**
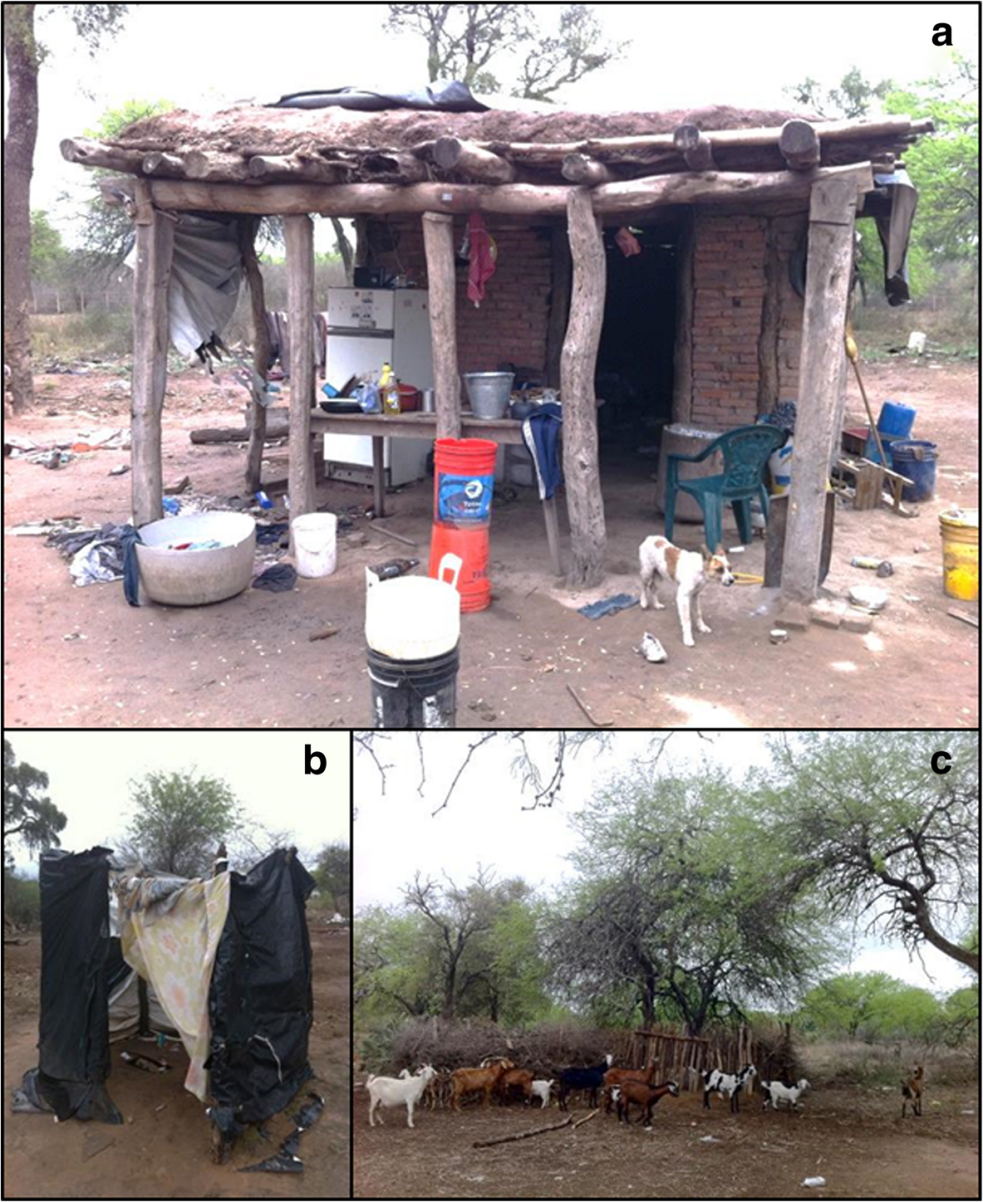
House from a rural lot in Añatuya, Santiago del Estero, Argentina (**a**) with details of the latrine (**b**) and animal pen (**c**)

Like many small localities in northern Argentina, the living conditions in the peri-urban and rural areas are characterized by a lack of water and sanitation [20, 33], and houses made out of adobe bricks with unimproved roofs and dirt floors (Fig. 2) [11, 24]. The livelihoods of most families are based on subsistence animal farming, government plans and day labourers [21, 26].

### Study design

This study was conducted as a community-based survey including all individuals older than 1 year-old for the rural population and children between 1–15 years-old, inclusive, for the peri-urban population. In total, three rural settlements (Lot 47, Lot 58 and Lot 27) and one peri-urban neighbourhood of Añatuya City (Colonia San Francisco) were included. After an initial meeting with the authorities, meetings were held directly with the community to explain the study and to give information about intestinal parasites and their prevention. The start day of the survey was communicated and on that given day, the research team visited the community house-by-house. All houses were georeferenced and a standardized questionnaire was used to collect data on all members of the household, socioeconomic variables and other details such as household characteristics and ownership of domestic and farm animals. Houses were considered to have unimproved roofs and walls if they were made out of material other than bricks or cement and they were considered to have unimproved floors if they were dirt floors. Unimproved water sources included rain, wells and waterholes which were all kept uncovered; improved water sources included the water supply system and bottled water. Unimproved bathrooms were defined as the absence of a latrine or latrines without a septic tank, and improved latrines as those with a septic tank or cesspit system. Most houses have only one room for sleeping so families with five members or more were considered to be overcrowded.

Stool containers were given to a responsible adult with verbal instructions on how to collect the faecal sample. Collection of the samples was arranged for the following day. The research team collected the samples house by house in a refrigerated container and transported them directly to the laboratory located in the city of Añatuya were they were frozen at -20 °C. The entire process was carried out within one week. Once all the samples were collected, the frozen samples were transported (frozen) to the city of Buenos Aires, directly to the Parasitology Laboratory of ANLIS of the National Ministry of Health where they were processed. The city of Buenos Aires is located approximately 928 km away from Añatuya (Fig. 1). All patients found to be infected with intestinal parasites were treated following national guidelines [34].

### Stool examination

In order to determine the presence of intestinal parasites, samples were processed by sedimentation [35] and two flotation techniques [36, 37]. The techniques chosen for this study are standard concentration techniques that increase the chances of detecting intestinal parasitic structures, including helminths and protozoans. These techniques fall into two categories: sedimentation is used mainly for the identification of eggs and cysts, while flotation is more appropriate for the detection of coccidian parasites [38].

#### Telemann sedimentation

Approximately 5–10 g of each faecal sample was homogenized with a 5% formalin saline solution (sodium chlorate, 40% formalin and dH_2_O) and then filtered through gauze placed in a funnel directly inside a 15 ml conical tube. Two millilitres of ether was added and the sample was centrifuged for 3–5 min at 1000–1500× *rpm*. After centrifugation, the supernatant was quickly decanted and the sediment was observed under a microscope between a slide and coverslip at 10× and 40×.

#### Sheather flotation

A drop of each concentrated sediment of the samples processed by the Telemann technique was homogenized with 1 ml of a sugar solution (saccharose, phenol crystals and dH_2_O) and after letting them stand for 3 min, a sample from the surface was placed between a slide and coverslip and observed under a microscope at 40×.

#### Willis flotation

Each faecal sample was homogenized with a super saturated salt solution in a plastic cup. Salt solution was then added so that one quarter of the cup was filled. A slide was placed on the edge of the cup and salt solution was added until the surface of the sample contacted the slide. The slide was left for 20 min, rapidly turned around, and then a coverslip was placed on top of the sample and observed under a microscope at 10×.

### Statistical analysis

Data was double-entered using Microsoft Excel (2010 Microsoft Corporation) and statistical analyses were performed using SPSS Statistics for Windows v.19.0. Population means, standard deviations (SD) and proportions with 95% confidence intervals (CI) were used to describe the study population and the prevalence of intestinal parasites. Chi-square test was used to compare proportions with a level of statistical significance of *P* < 0.05. Odds ratios, *z*-values and 95% CI were computed in order to determine associations between household characteristics and presence or absence of intestinal parasites. A multivariate analysis was also performed using those variables that had a *P-*value less than 0.025. Therefore, the logistic binary regression using the presence/absence of intestinal parasites as the dependent variable was performed including the following eight independent variables: age (in years), gender, overcrowding, walls, floor, electricity source, animal farming and informal slaughter.

## Results

### Study population

The neighbourhood of Añatuya included in this study, named Colonia San Francisco, is a peri-urban neighbourhood whose inhabitants make a living mainly through the traditional manufacturing of bricks. The rural settlements (Lot 27, Lot 47 and Lot 58) evaluated are located on both sides of provincial route 7 to the east of Añatuya, approximately 40 to 50 km away (Fig. 1). Participation, measured as the number of individual containers returned with a sample divided by the number of containers given, was high in both the rural settlements and the peri-urban neighbourhood of Añatuya: 74.8% (101/135) for Lot 47, 80.1% (173/216) for Lot 58, 74.2% (46/62) for Lot 27, and 79.8% (150/188) for Colonia San Francisco.

In total, 470 individuals from 143 households provided stool samples. Descriptive characteristics of the participants and prevalence of intestinal parasites are detailed in Table 1. The mean age was not significantly different between the rural lots where the entire community was included in the study, except for the comparison between Lot 47 and Lot 58 due to the large number of children present in Lot 58 which lowered the mean age of the community (*P* < 0.05). The mean age in Colonia San Francisco was 8 years since only children between 1–15 years-old were included. The distribution by gender did not vary significantly between the rural lots or in comparison with the peri-urban neighbourhood (*P* > 0.05). Most children from all four localities were school or pre-school-aged. On the other hand, many adults in the three rural lots did not finish elementary school; this was particularly marked in Lot 58 where only 25 out of 82 adults (30.5%) finished primary school.

**Table 1:** Descriptive characteristics of participants and prevalence of intestinal parasites

	Rural settlements			Total	Peri-urban neighbourhood	Total
	Lot 47 (*n* = 101)	Lot 58 (*n* = 173)	Lot 27 (*n* = 46)	Rural area (*n* = 320)	Colonia San Francisco (*n* = 150)	Rural and peri-urban (*n* = 470)
Age, mean ± SD	32.7 ± 21.8	21.4 ± 18.1	27.4 ± 21.7	25.8 ± 20.5	8.0 ± 4.0	20.0 ± 19.1
Range	2–86	1–72	1–83	1–83	1–15	1–83
Gender, *n* (%)						
Female	42 (41.6)	83 (48.0)	26 (56.5)	151 (47.2)	83 (55.3)	234 (50.2)
Male	59 (58.4)	90 (52.0)	20 (43.5)	169 (52.8)	67 (44.7)	236 (49.8)
Finished elementary school, *n* (%)	41 (40.6)	25 (14.5)	25 (54.3)	91 (28.4)	0 (0)	91 (19.4)
Currently in school, *n* (%)	24 (23.8)	68 (39.3)	12 (26.1%)	104 (32.5)	124 (82.67)	228 (48.5)
Prevalence of protozoans, *n* (%) [95% CI]	17 (16.8) [10.8–25.3]^a^	14 (8.1) [4.9–13.1]^b,c,e^	1 (2.2) [0.4–11.3]^f^	32 (10.0) [7.2–13.8]	23 (15.3) [10.4–22.0]^f,h,i,j^	55 (11.7) [9.1–14.9]
*Entamoeba coli*	3 (3.0) [1.0–8.4]	7 (4.0) [2.0–8.1]	0	10 (3.1) [1.7–5.7]	2 (1.3) [0.4–4.7]	12 (2.6) [1.5–4.4]
*Blastocystis hominis*	1 (1.0) [0.2–5.4]	1 (0.58) [0.1–3.2]	0	2 (0.63) [0.17–2.3]	1 (0.67) [0.1–3.7]	3 (0.64) [0.22–1.9]
*Chilomastix mesnili*	5 (5.0) [2.1–11.1]	0	1 (2.2) [0.4–11.3]	6 (1.9) [0.87–4.0]	5 (3.3) [1.4–7.6]	11 (2.3) [1.9–4.1]
*Giardia lamblia*	8 (7.9) [4.1–14.9]	6 (3.5) [1.6–7.4]	1 (2.2) [0.4–11.3]	15 (4.7) [2.9–7.6]	19 (12.7) [8.2–18.9]	34 (7.2) [5.2–9.9]
*Endolimax nana*	3 (3.0) [1.0–8.4]	1 (0.58) [0.1–3.2]	0	4 (1.3) [0.49–3.2]	0	4 (0.85) [0.33–2.2]
Prevalence of helminth parasites, *n* (%) [95% CI]	3 (3.0) [1.0–8.4]	26 (15.0) [10.5–21.1]^c,d,e^	1 (2.2) [0.4–11.3]	30 (9.4) [6.7–13.1]	32 (21.3) [15.5–28.6]^g,h,i,j^	52 (11.1) [8.5–14.2]
*Enterobius vermicularis*	1 (1.0) [0.2–5.4]	9 (5.2) [2.8–9.6]	1 (2.29 [0.4–11.3]	11 (3.4) [1.9–6.1]	16 (10.7) [6.7–16.6]	27 (5.7) [4.0–8.2]
Hookworm	2 (2.0) [0.5–6.9]	0	0	2 (0.63) [0.17–2.3]	0	2 (0.43) [0.12–1.6]
*Hymenolepis nana*	0	21 (12.1) [8.1–17.9]	0	21 (6.6) [4.3–9.8]	18 (12.0) [7.7–18.2]	39 (8.3) [6.1–11.1]

^a^Two co-infections *G. lambia* + *C. mesnili* and one co-infection *C. mesnili* + *E. coli* + *E. nana*

^b^One co-infection *B. hominis + E. coli*

^c^One co-infection *E. vermicularis + E. coli*

^d^Four co-infections *E. vermicularis* + *H. nana*

^e^One co-infection *G. lamblia* + *H. nana*

^f^One co-infection *G. lamblia* + *C. mesnili*

^g^One co-infection *E. vermicularis* + *H. nana*

^h^One co-infection *G. lamblia* + *H. nana*

^i^Three co-infections *G. lamblia* + *C. mesnili* + *H. nana*

^j^One co-infection *E. vermicularis* + *H. nana* + *C. mesnili*

*Abbreviations*: *SD* standard deviation, *CI* confidence interval, *PSAC* preschool-aged children

### Prevalence of intestinal parasites

The overall prevalence of intestinal parasites was 22.6% (106/470); 11.7% (55/470) for protozoans, 11.1% (52/470) for helminths and 2.1% (10/470) mixed infections. Moreover, 4.5% (21/470) of the participants were polyparasitized. The most common co-infections found were *Hymenolepis nana* + *Enterobius vermicularis* (*n* = 5) and *H. nana* + *Giardia lamblia* (*n* = 5). The locality with the highest prevalence for protozoans was Lot 47 (16.8%; 17/101), while the prevalence of helminths was the highest in the peri-urban neighbourhood of Colonia San Francisco (21.3%; 32/150). The most prevalent protozoan was *G. lamblia* (7.2%; 34/470) and it was present in all the localities, while the most prevalent helminth was *H. nana* (8.3%; 39/470) which was only present in Lot 58 and Colonia San Francisco. The only STH found in the 470 samples was hookworm, with only 2 cases from Lot 47 (2/470; 0.43%). As expected, intestinal parasites were significantly more prevalent in pre-school (*P* < 0.001) and school-aged children (*P* < 0.001) than in adults (Table 2). There was no association with gender but there was an association with respect to level of education in adulthood since the infection was significantly different between adults that finished elementary school (*P* < 0.001). Nonetheless, there was also a significant difference between adults that did not finish elementary school (*P* < 0.001).

**Table 2:** Characteristics of participants classified by presence or absence of intestinal parasites

Characteristics	No. (%) of positive participants (*n* = 106)	No. (%) of negative participants (*n* = 364)	*χ* ^2^	*P*	Total no. (%) of participants (*n* = 470)
Age group (years)					
1–5	30 (28.3)	65 (17.9)	5.49	**0.019**	95 (20.2)
6–15	61 (57.5)	134 (36.8)	14.46	**<0.001**	195 (41.5)
16–26	8 (7.6)	44 (12.1)	1.68	0.195	52 (11.1)
27–67	7 (6.6)	109 (29.9)	23.95	**<0.001**	116 (24.7)
≥ 68	0	12 (3.3)	3.58	0.058	12 (2.5)
Gender					
Female	56 (52.8)	178 (48.9)	0.36	0.475	234 (49.8)
Male	50 (47.2)	186 (51.1)			236 (50.2)
Education					
≤ 3 years-old	11 (10.5)	39 (10.7)	0.003	0.953	50 (10.6)
In pre-school	17 (16.0)	20 (5.5)	12.46	**<0.001**	37 (7.9)
In elementary school	60 (56.6)	119 (32.7)	19.84	**<0.001**	179 (38.3)
In high school	1 (0.9)	11 (3.0)	1.47	0.225	12 (2.3)
Finished elementary school	9 (8.5)	82 (22.5)	10.29	**0.001**	91 (19.4)
Incomplete elementary school	7 (6.6)	72 (19.8)	10.20	**0.001**	79 (16.8)
Finished high school	0	2 (0.6)	0.64	0.424	2 (0.4)
No formal education	1 (0.9)	19 (5.2)	3.74	0.053	20 (4.3)

Bold indicates the relationship was statistically significant *P* < 0.05

### Household characteristics

A questionnaire was filled out by a member of the team together with a responsible adult from each of the households. The main characteristics from the houses of each locality included in the study are detailed in Table 3. The most common livelihoods were animal farming in Lot 47 (96.9%; 31/32) and Lot 27 (100%; 15/15), day labourers in Lot 58 (57.3%; 47/82) and workers in traditional brick factories for Colonia San Francisco (78.7%; 48/61. Nonetheless, most inhabitants did not have formal jobs and in both the rural lots and the peri-urban neighbourhood, many adults received government pensions or retirement allowances (41.3%; 59/143) and many families received child allowances from the government (46.2%; 66/143). The mean number of inhabitants per household was similar in both the rural settlements and the peri-urban neighbourhood of Añatuya, although the number of families with five members or more was highest in Colonia San Francisco (62.3%; 38/61) and Lot 58 (65.7%; 23/35). These households were considered to be overcrowded since, in general, the structures had only one room for sleeping. Nonetheless, there was no association between the presence of intestinal parasites and overcrowding.

**Table 3:** Household characteristics of participants

Characteristics	Rural settlements			Total	Peri-urban neighbourhood	Total
	Lot 47 (*n* = 32)	Lot 58 (*n* = 35)	Lot 27 (*n* = 15)	Rural area (*n* = 82)	Colonia San Francisco (*n* = 61)	Rural and peri-urban (*n* = 143)
Primary income/livelihood, *n* (%)	31 (96.9) animal farm; 19 (59.4) day labourer	24 (68.6) day labourer; 20 (57.1) animal farm	15 (100) animal farm; 10 (62.5) pension/retirement	67 (81.7) animal farm; 47 (57.3) day labourer; 39 (47.6) pension/retirement; 34 (41.5) social plans	48 (78.7) day labourer; 32 (52.5) social plans	95 (66.4) day labourer; 91 (63.6) animal farm; 66 (46.2) social plans; 59 (41.3) pension/ retirement
Mean no. of inhabitants per household	4.4	6.2	4.5	5.2	5.7	5.4
Houses with 5 inhabitants or more, *n* (%)	13 (40.6)	23 (65.7)	8 (53.3)	44 (53.7)	39 (63.9)	83 (58.0)
Unimproved roof, *n* (%)	30 (93.8)	35 (100)	11 (73.3)	76 (92.7)	37 (61.7)	113 (79.0)
Unimproved walls, *n* (%)	12 (37.5)	30 (85.7)	8 (53.3)	50 (61.0)	2 (3.3)	52 (36.4)
Unimproved floor, *n* (%)	27 (84.4)	33 (94.3)	13 (86.7)	73 (89.0)	7 (11.7)	80 (55.9)
Main energy source, *n* (%)	17 (53.1) generator	18 (51.4) none	8 (53.3) generator	30 (36.6) generator	55 (91.7) electricity network	55 (38.5) electricity network; 30 (21.0) generator
Main drinking water source, *n* (%)	19 (59.4) water truck	34 (97.1) well	13 (86.7) well	53 (64.6) well	42 (68.9) water supply system	53 (37.1) well; 42 (29.4) water supply system
Main cooking water source, *n* (%)	19 (59.4) water truck	34 (97.1) well	12 (80.0) well	52 (63.4) well	42 (68.9) water supply system	52 (36.4) well; 42 (29.4) water supply system
Main source of water for bathing/handwashing, *n* (%)	19 (59.4) water truck	35 (100) well	13 (86.7) well	56 (68.3) well	42 (68.9) water supply system	56 (39.2) well; 42 (29.4) water supply system
Unimproved latrine, *n* (%)	17 (53.1); 15 (46.9) lack latrine	17 (48.6); 18 (51.4) lack latrine	15 (100)	49 (59.8); 33 (40.2) lack latrine	21 (35.0); 9 (15.0) lack latrine	70 (49.0); 42 (29.4) lack latrine
Presence of kitchen garden, *n* (%)	9 (28.1)	6 (17.1)	13 (86.7)	28 (34.1)	2 (3.3)	30 (21.0)
Presence of animal pen, *n* (%)	26 (81.3)	22 (62.9)	13 (86.7)	61 (74.4)	13 (21.7)	74 (51.7)

As far as the houses themselves, most in the rural lots had unimproved roofs (92.7%; 76/82), walls (61.0%; 50/82) and floors (89.0%; 73/82), while the houses in Colonia San Francisco were made out of bricks and cement, except for the roofs which were largely unimproved (61.7%; 37/61). On the other hand, the rural lots had significantly higher percentages of unimproved roofs, walls and floors in comparison to Colonia San Francisco (*P* < 0.05). Despite this, many of the houses in both rural lots and Colonia San Francisco had either unimproved latrines (49.0%; 70/143) or none at all (29.4%; 42/143), although this was more marked in the rural lots. Concerning amenities, only Colonia San Francisco was connected to the electricity network and the water supply system.

The highest prevalence of waterborne parasites, amoebas and *Giardia*, was seen in Lot 47 (16.8%) and Colonia San Francisco (15.3%), regardless of the source of water for drinking. The majority of the households in Lot 47 buy water from Añatuya (59.4%; 19/32) and most of the houses in Colonia San Francisco (68.9%; 42/61) are supplied by the water system. On the other hand, the highest percentages of parasites transmitted by the faecal-oral route and close contact, mainly *H. nana* and *E. vermicularis*, were seen in Lot 58 (15.0%; 26/173) and Colonia San Francisco (21.3%; 32/150). These results coincide with a greater percentage of households with five or more family members stated above, which facilitates transmission, but also with the amount of children under 14 years of age. In Lot 58, 80% (28/35) of the households had children under 14 years-old, while in Colonia San Francisco only houses with children under 14 years-old were included.

When analysing the association between household characteristics and the presence or absence of intestinal parasite infection (Table 4), there were significant associations with informal slaughter of animals, the walls and the floors of the house. The odds of having intestinal parasites was reduced by 37% in those individuals with improved walls (0.635, 95% CI: 0.406–0.993, *P* = 0.046). Unexpectedly, slaughtering animals informally in the household (0.505, 95% CI: 0.326–0.782, *P* = 0.002) and having unimproved floors (0.593, 95% CI: 0.381–0.924, *P* = 0.021), also slightly reduced the odds of infection. Moreover, having electricity lowered the odds of being infected by 36% (0.642, 95% CI: 0.408–1.012), although this difference did not reach statistical significance (*P* = 0.0563).

**Table 4:** Household and livelihood characteristics and their association with the presence or absence of intestinal parasites

Characteristics (risk factor = 1)	Presence of IP (*n* = 106)	Absence of IP (*n* = 364)	OR (95% CI)	*P*-value
Animal farming				
Yes (1)	65 (61.7)	250 (68.6)	0.723 (0.461–1.133)	0.157
No	41 (38.3)	114 (31.4)		
Informal slaughter				
Yes (1)	48 (45.8)	226 (62.0)	0.505 (0.326–0.782)	0.002
No	58 (54.2)	138 (38.0)		
Kitchen garden				
Yes (1)	11 (10.3)	38 (10.5)	0.993 (0.489–2.018)	0.985
No	95 (89.7)	326 (89.5)		
Unimproved roof (1)	83 (78.5)	298 (81.8)	0.799 (0.469–1.362)	0.410
Improved roof	23 (21.5)	66 (18.2)		
Unimproved walls (1)	63 (59.8)	254 (69.7)	0.635 (0.406–0.993)	0.046
Improved walls	43 (40.2)	110 (30.3)		
Unimproved floor (1)	40 (38.3)	184 (50.4)	0.593 (0.381–0.924)	0.021
Improved floor	66 (61.7)	180 (49.6)		
Unimproved water source (1)	60 (56.1)	217 (59.8)	0.884 (0.571–1.369)	0.579
Improved water source	46 (43.9)	147 (40.2)		
Unimproved latrine (1)	85 (80.4)	287 (78.8)	1.086 (0.633–1.863)	0.765
Improved latrine	21 (19.6)	77 (21.2)		
Electricity network as main energy source				
No (1)	66 (62.3)	262 (72.0)	0.642 (0.408–1.012)	0.056
yes	40 (37.7)	102 (28.0)		
Overcrowding^a^				
Yes (1)	83 (78.3)	264 (72.5)	1.367 (0.816–2.290)	0.235
No	23 (21.7)	100 (27.5)		

^a^Overcrowding, households with 5 or more inhabitants per house

Significant *P*-values set at < 0.05

*Abbreviations*: *CI* confidence interval, *IP* intestinal parasites, *OR* odds ratio

All values are *n* (%). Reference group marked as OR = 1

A multivariate analysis was performed in order to further analyse the associations (Table 5). The model obtained using logistic binomial regression was valid, Chi-square test (8, *n* = 470): *χ*^2^ = 45.80, *P* < 0.001, and between 9.3–14.1% of the variance in the dependent variable being explained by the model. The percent accuracy in classification (PAC) of the model was 77.0%. In this multivariate analysis, only age and electricity source had predictive capacity. In the model, age was negatively associated with intestinal parasites (*P* < 0.001), while not being connected to the electricity network increased the odds of having IP by 2.54 (*P* = 0.026).

**Table 5:** Results of the multivariate analysis performed through logistic binary regression using the presence/absence of intestinal parasites as the dependent variable

Variable	AOR (95% CI)	*P*-value
Age (years)	0.951 (0.931–0.970)	<0.001
Gender (male)	0.883 (0.560–1.392)	0.591
Overcrowding (no)^a^	0.824 (0.472–1.438)	0.495
Animal farming (no)	0.976 (0.544–1.754)	0.936
Walls (unimproved)	0.853 (0.385–1.891)	0.696
Floor (unimproved)	0.604 (0.321–1.137)	0.118
Electric network (no)	2.540 (1.119–5.764)	0.026
Informal slaughter (yes)	1.650 (0.865–3.149)	0.129

^a^Overcrowding, households with 5 or more inhabitants per house

*Abbreviations*: *AOR* adjusted odds ratio, *CI* confidence interval

Significant values set at < 0.05

## Discussion

Most intestinal parasites have a cosmopolitan distribution and are present in both rural and peri-urban areas, especially those that are acquired *via* waterborne transmission or through direct contact [39]. Other intestinal parasites, such as STH, are more prevalent in tropical and sub-tropical areas of the world, probably due to their need of passage through the soil, making them more sensitive to the external environment [12, 40, 41]. Living conditions are also associated with the presence of intestinal parasites, including STH, since they are excreted in the faeces and therefore the lack of improved sanitation and access to clean water are key factors [1, 9, 13]. Nonetheless, even in areas appropriate for their development and transmission, STH are heterogeneously geographically distributed and aggregated in the population [42, 43]. In Argentina, STH have been detected mainly in northern provinces that have a sub-tropical humid climate, with the most prevalent STH being hookworm and *S. stercoralis* [28]. Most of this data is from published point studies [17, 24, 33, 44, 45] while a comprehensive map of the prevalence and distribution of STH is lacking.

The overall prevalence of intestinal parasites found in the present study was 22.6%. The percentage of individuals parasitized and polyparasitized was low in comparison to studies conducted in other areas of northern Argentina [6, 11, 17, 18, 20, 21, 25, 26]. Hyperendemic areas for hookworm [26], *A. lumbricoides* [17] and *S. stercoralis* [25] have been detected in communities similar to those included in this study, and although the living conditions seem to be appropriate for the transmission of STH, only two cases of hookworm were found and no other STH detected. The population included in this study is from an area of the country located in the semiarid sub-region of the Great Chaco ecoregion. Moreover, the sediments around this area are thin and not very permeable, with a high accumulation of salt [32]. We believe this is the reason why the presence of STH was negligible. The influence of soil type on the distribution of STH has been observed in other studies [41, 46, 47] and future studies in Argentina should evaluate this relationship.

In our study, parasitism was not associated to gender as in other studies [48–50], although we did detect a negative significant association with age, with a decrease in risk as age increases, which has also been observed by others [48, 51]. An association with level of education and overcrowding was not detected and this also coincides with other studies [50, 52]. Most of the parasites found in the present study are transmitted though water, although an association between the source of drinking water and presence of infection was not significant in agreement with previous findings [46, 50]. Both Lot 47 and Colonia San Francisco had the highest prevalence of amoebas and *G. lamblia*, 16.8 and 15.3%, respectively. This is regardless of the source of drinking water: 59.4% of the houses from Lot 47 bought their water from Añatuya and 68.9% of the houses in Colonia San Francisco were supplied by the water system, which means that most of the inhabitants of Lot 47 and Colonia San Francisco had the same source of water.

Añatuya’s water for the supply system comes from two sources: (i) subterranean water extracted by the company Aguas de Santiago in El Simbolar and carried by a 180 km aqueduct; and (ii) water from the Salado River that is purified through reverse osmosis in a plant located 5 km southwest of Añatuya. The water from both sources is stored in a deposit located in Añatuya and distributed through the piped water supply system. This does not seem to guarantee the quality of the water used in the system, and somewhere along the line (i.e. at origin, through transport or during storage) the water is contaminated. Another source of infection could be a lack of hygiene, since studies have seen an association between infection with intestinal parasites and handwashing as well as nail clipping [14–16].

On the other hand, Lot 58 and Colonia San Francisco were the localities with the highest prevalence of helminth parasites transmitted by the faecal-oral route and through direct contact (15.0 and 21.3%, respectively). The most prevalent helminth in both localities was *H. nana* (21 and 18%, respectively). Few epidemiological studies focus on this parasite even though moderate to high prevalences have been detected in Argentina [21, 26, 31] and in other countries the Americas [48, 53]. *Enterobius vermicularis* was also detected in this study but the specific method for its diagnosis was not used since it was not the focus of this study, probably leading to underestimation of the prevalence of this parasite. Both these localities had the highest proportion of children and the greatest amount of households with overcrowding, which favours transmission of these parasites [16]. Moreover, the lack of latrines or the use of unimproved latrines not only favours environmental contamination but also leads to a lack of hygiene since after defecation, individuals do not have an appropriate place to wash their hands.

In this study, some of the household characteristics were significantly associated with the presence of intestinal parasites in the univariate analysis but not in the multivariate one. The presence of unimproved walls favoured the presence of IP and this could be used as a proxy for socioeconomic status. On the other hand, the presence of an improved floor seemed to favour transmission of IP. This seems to be counterintuitive since one would think that having cement floors would prevent the transmission of IP. The significant association between informal slaughtering and the absence of IP might also be interpreted as a proxy for socioeconomic status, since not all families are able to access meat for consumption. Some studies have detected contrary effects of having electricity [54, 55] and, in our study, not being connected to the electricity network increased the odds of having IP by 2.54, reaching significance in the multivariate analysis.

In general, the household characteristics of both the rural and peri-urban localities included in this study were very similar with respect to the type of construction, water and sanitation. The livelihood and level of education of the population was also very similar between the localities. These generally similar characteristics between those that are parasitized and those that are not may be influencing the power of the statistical analysis. In order to be able to obtain significant associations it might be necessary to study more distinct populations [50]. It is important, when considering these results, to point out the some of the limitations of this study. First, a single stool sample was used to determine the prevalence. Secondly, the samples were frozen in order to preserve them for future molecular studies since the laboratory used for the analysis was quite distant. Because the samples were frozen, we could not perform a faecal culture technique for the specific diagnosis of *S. stercoralis*. Therefore, the actual prevalence of infection by intestinal parasites might be underestimated and this could have also impacted our statistical power to detect associations between infection and household characteristics.

## Conclusions

The prevalence of intestinal parasites in the study population was 22.6%. Out of the 106 individuals infected, 91 (85.8%) were children between the ages of 1 and 15 years, and there was a significant inverse association between age and parasitism. Out of all the household characteristics studied, only the main source of electricity was significantly associated with the presence of IP. A total of 11.7% of the parasites detected are transmitted through water, evidencing the need to improve quality control in water facilities and access to improved sanitation in order to avoid contamination of the stored water. The most prevalent helminth parasite found was *H. nana* (8.3%), a parasite that is generally not given much attention, and the presence of STH was negligible.

## Abbreviations

CI: Confidence interval
DALYs: Disability-adjusted life years
IP: Intestinal parasites
LAC: Latin America and the Caribbean
MDA: Mass drug administration
NTDs: Neglected tropical diseases
PAC: Percent accuracy classification
SAC: School aged children
SD: Standard deviation
STH: Soil-transmitted helminths
WASH: Water, sanitation and hygiene
WHA: World Health Assembly
WHO: World Health Organization
